# Lipidomic profiling reveals distinct differences in plasma lipid composition in overweight or obese adolescent students

**DOI:** 10.1186/s12902-021-00859-7

**Published:** 2021-10-13

**Authors:** Ruili Yin, Xiaojing Wang, Kun Li, Ke Yu, Longyan Yang

**Affiliations:** Beijing Key Laboratory of Diabetes Prevention and Research, Center for Endocrine Metabolic and Immune Diseases, Beijing Luhe Hospital, Capital Medical University, Beijing, 101149 China

**Keywords:** Lipidomic, Obesity, Overweight, Adolescence

## Abstract

**Introduction:**

The relationship between dyslipidemia and obesity has been widely reported, but the global lipid profiles associated with the development of obesity still need to be clarified. An investigation into the association between the lipidomic plasma profile and adolescent obesity may provide new insights into the development of obesity.

**Methods:**

Mass spectrometry coupled with liquid chromatography was applied to detect the global lipidome in the fasting plasma from 90 Chinese adolescents, including 34 obese adolescents, 26 overweight adolescents, and 30 adolescents with a normal body mass index (BMI). All participants underwent anthropometric measurements by using InBody. Clinical biochemical indicators were measured by Cobas Elecsys.

**Results:**

Both qualitative and quantitative analyses revealed a gradual change in plasma lipid features among obese students, exhibiting characteristics close to overweight students, but differing significantly from normal students. Compared with normal and overweight students, levels of triglyceride (TG), 18-hydroxycortisol, isohumulinone A, and 11-dihydro-12-norneoquassin were up-regulated in the obese group, while phosphatidylcholine (PC), phosphatidylethanolamine (PE), lysoPC (LPC), lysoPE (LPE), and phosphatidylinositol (PI) were significantly down-regulated in the obese group. Then, we conducted Venn diagrams and selected 8 significant metabolites from the 3 paired comparisons. Most of the selected features were significantly correlated with the anthropometric measurements.

**Conclusions:**

This study demonstrated evidence for a relationship between the eight significant metabolites with obese adolescents. These lipid features may provide a basis for evaluating risk and monitoring the development of obesity.

**Supplementary Information:**

The online version contains supplementary material available at 10.1186/s12902-021-00859-7.

## Background

A high prevalence of obesity and metabolic syndrome can now be observed in both adults and young people. These phenomena affect 380 million children and adolescents worldwide [1]. Childhood obesity has a significant impact on both physical and psychological health [2]. It could lead to metabolic, pulmonary, orthopedic, neurological, cardiovascular, hepatic, and menstrual disorders [3]. WHO defines adolescence as a period of growth and development between the ages of 10 and 19 years after childhood and before adulthood. It is one of the important transition periods in the life cycle and is characterized by a large amount of growth and change, second only to infancy.

It is believed that adolescent and childhood obesity have reached epidemic levels [1], and about 17% of children are facing obesity problems in the United States [2]. The increased prevalence of overweight and obesity in children and adolescents has been observed in several countries, and weight gain is an independent predictor for metabolic syndrome development, although it is not seen in all obese individuals. Metabolic syndrome is defined as the presence of a combination of risk factors for cardiovascular disease and type 2 diabetes, including obesity, dyslipidemia, hypertension and glucose intolerance [4]. The above conditions, although seen more frequently in adults, can manifest at earlier ages [5, 6]. Therefore, the diagnosis of the possible presence of obesity at early ages, accompanied by control interventions, should have a favorable impact on the health of adult people and the prevention of cardiovascular outcomes.

Previous studies have demonstrated that considerable alterations in lipid metabolism and consequently marked changes in lipid profile are associated with the onset and progression of obesity-related complications. By comparing the metabolomics characteristics of obesity, Newgard et al. revealed resistance-related BCAA-related metabolite characteristics, and the accompanying specific increase in C3 and C5 carnitine levels, which indicated an increase in BCAA catabolism [7]. Longitudinal lipidomics studies in children have shown that maternal obesity increases the risk of offspring obesity, which is marked by a long-term change in plasma ceramide levels [8]. To study the changes of metabolites in blood lipids by lipidomics, caloric restriction and the improvement of metabolic syndrome following fish oil intake were predicted, and potential lipid metabolites were identified [9]. Pawelzik et al. performed lipidomic analysis of urine samples from obese people and identified a relationship between urinary prostaglandin levels and obesity-related dyslipidemia, abdominal obesity, and insulin resistance [10]. Péter Pikó et al. carried out a more sophisticated lipidomic analysis by introducing a stepwise regression analysis and LSR calculation. In this way, we identified four and three key lipid species showing a strong significant positive (PE P-16:0/20:3, TG 20:4_33:1, TG 22:6_36:4, TG 18:3_33:0) and negative (Hex-Cer 18:1;O2/22:0, LPC 18:2, PC 18:1_18:1) association with BMI, respectively [11]. These regularly determined laboratory parameters provide information on lipid disturbances in general, today it is widely accepted that characterization of the full spectrum of obesity-induced changes in lipid metabolism, however, a detailed analysis of the children plasma lipidome, is required to create attractive hypotheses on the patho-mechanisms of obesity and identify sensitive predictive and prognostic biomarkers, as well as targets to their prevention and therapy [12].

Conventional data-dependent acquisition (DDA) mass spectrometry (MS) mode has been widely used in lipidomic studies, where parameters are detected to minimize duplicate precursor ions and can be optimized to identify complex lipid molecules [13]. However, DDA performance has some inherent limitations, such as a limited dynamic range, a bias against highly abundant ions, and long duty cycles with increasing sample complexity. A data independent acquisition (DIA) strategy was recently developed to alleviate the limitations of the DDA model [14], which improves detection sensitivity and analytical reproducibility. However, the independent data acquisition method is not easy to apply to lipidomics because the annotation of MS features and the estimation of the false discovery rate in large and complex lipid data sets require more sophisticated software and integrated reference databases [15].

Here, we conducted a non-targeted lipidomic analysis of 90 Chinese adolescent students, including 30 obese students, 26 overweight students, and 34 students with normal BMI, using DIA-based liquid chromatography–tandem mass spectrometry (LC-MS/MS). By using statistical business and in-house software to analyze the highly complex data sets, we demonstrated that compared with overweight and normal students, obese students in China have significant changes in lipids in their plasma. In addition, we identified several lipid characteristics, including TG, 18-hydroxycortisol, isohumulinone A, and 11-dihydro-12-norneoquassin, PC, PE, LPC, LPE, and PI, which are potential indicators for predicting obesity risk.

## Materials and methods

### Study population

Nighty teenagers from junior middle school took part in the study (Beijing 9th Middle School). In addition, the principal’s approval was obtained before visiting the school. During the first visit, a consent form with research information was distributed to the students. We encouraged the students to bring back the consent form the next morning. On the next visit to the school, the children who brought back signed consent forms were screened for inclusion. The trial was approved by the Ethics Committee at the Luhe Hospital affiliated with Capital Medical University. A total of 100 students participated in the study. Of these, 10 students refused to continue their cooperation because of different reasons (such as parents’ dissatisfaction with blood sampling, interference with curricula, and fear of blood sampling). Finally, 90 students were enrolled in the study. The inclusion criteria were age between 12 to 13, the consent to participation of the students and their parents, lack of illnesses affecting weight, non-use of drugs affecting weight, and not having diet and exercise programs interfering with weight. The volunteers with serious diseases, any musculoskeletal diseases and special diets or recent weight changes were excluded.

### Data collection and anthropometric measurement

The participants underwent anthropometric measurements by using InBody 770 (InBody Co. Ltd., Seoul, Republic of Korea). We evaluated the collected data from the anthropometric measurements statistically and graphically in Microsoft Office Excel 2010 (Los Angeles, CA, USA). In this study, blood was collected to coagulate at 4 °C and the serum was separated by centrifugation for 15 min at 3000 rpm. Serum TSH, FT4 and FT3 were tested with an electrochemiluminescence immunoassay (ECLIA) using an Abbott Architect I2000 (Abbott Diagnostics, Abbott Park, IL, USA). Clinical biochemical indicators were measured by a Cobas Elecsys 601 (Roche Diagnostics, Switzerland). The children are grouped according to the BMI Z-score of WHO child growth standards [16], and the age is the exact value. Those with a Z > 1 are defined as overweight group, those with a Z > 2 are defined as the obese group, and those with a Z ≤ 1 are defined as normal and thin groups.

To delineate global lipidomic profiles in Chinese overweight and obese adolescents, BMI and body fat percentage together with the corresponding clinical and phenotypic data were collected from the 3 groups in Beijing, China (Additional file 1).

### Liquid chromatography–tandem mass spectrometry (LC-MS/MS)

Lipids were extracted from individual plasma samples and then injected into the mass instrument in both positive and negative modes, with pooled extraction quality control (QC) samples at certain intervals. In this project, the advanced mass spectrometer Xevo G2-XS QTOF (Waters, UK) was used for mass spectrometry data collection, and the commercial software PROGENESIS QI (Version 2.2) (Waters, UK) and the independently developed metabonomics R software package metaX were used for statistical analysis of the mass spectrometry data, wherein metabolite identification was based on the databases HMDB and LipidMaps [17]. Univariate and multivariate analyses were conducted using R statistics software to identify and evaluate the significant metabolites among the groups.

### Metabolites extraction method

First, 40 μL of each sample was added to the corresponding 96-well plate; 120 μL of pre-cooled isopropyl alcohol was added, shaken and mixed for 1 min, and then placed at − 20 °C for 2 h or overnight, followed by centrifugation at 4000 g at 4 °C for 30 min. We placed the supernatant in a new 96-well plate and diluted it with 225 μL of lipid complex solution (isopropanol: acetonitrile: water = 2: 1: 1). Then, 20 μL of each sample was mixed with the QC sample and 60 μL of the supernatant was transferred to a 96-well microtiter plate, sealed, and tested on the machine.

### LC-MS parameters

All samples were acquired by the LC-MS system followed machine orders. Firstly, all chromatographic separations were performed using an ultra-performance liquid chromatography (UPLC) system (Waters, UK). An ACQUITY UPLC CSH C18 column (100 mm × 2.1 mm, 1.7 μm, Waters, UK) was used for the separation. The column was maintained at 55 °C. The flow rate was 0.4 mL/min and the mobile phase consisted of solvent A (ACN: H_2_O = 60:40, 0.1% formate acid and 10 mM ammonium formate) and solvent B (IPA: ACN = 90:10, 0.1% formate acid and 10 mM ammonium formate). Gradient elution conditions were set as follows: 0 ~ 2 min, 40–43% phase B; 2.1 ~ 7 min, 50–54% phase B; 7.1–13 min, 70–99% phase B; 13.1–15 min, 40% phase B. The injection volume for each sample was 10 μL.

### Mass spectrometer description

A high-resolution tandem mass spectrometer Xevo G2 XS QTOF (Waters, UK) was used to detect metabolites eluted from the column. The Q-TOF was operated in both positive and negative ion modes. For positive ion mode, the capillary and sampling cone voltages were set at 3.0 kV and 40.0 V, respectively. For negative ion mode, the capillary and sampling cone voltages were set at 2 kV and 40 V, respectively. The mass spectrometry data were acquired in Centroid MSE mode. The TOF mass range was from 100 to 2000 Da in positive mode and 50 to 2000 Da in negative mode. The survey scan time was 0.2 s. For the MS/MS detection, all precursors were fragmented using 19–45 eV, and the scan time was 0.2 s. During the acquisition, the LE signal was acquired every 3 s to calibrate the mass accuracy. Furthermore, in order to evaluate the stability of the LC-MS during the entire acquisition, appropriate standards were run and a quality control sample (a pool of all samples) was also acquired after every 10 samples.

### Nomenclature of metabolites

For example, 6.10_ 861.5490 m/z was the retention time_ charge mass ratio. The identification results (PC (15:0/0:0), PE (18:0/0:0), LPC (15:0), and LPE (0:0/18:0)) were obtained by comparing the retention time and charge mass ratio information of the collected ions with the information in the KEGG and HMDB databases.

### Statistics analysis

All data was tested with chi-square tests first, then Tukey HSD analysis was applied if it met the normal distribution and the Kruskal-Wallis test was applied if not, and Dunn’s post hoc tests followed by pairwise comparisons were performed. The mean differences in metabolites were analyzed by one-way ANOVA, Bonferroni’s multiple comparison test analysis. Associations between lipid and clinical or anthropometric parameters were determined by Pearson correlation coefficients by GraphPad Prism 7. A *P*-value < 0.05 was considered significant.

## Results

### Assessment of clinical characteristics and plasma lipidomic features

The clinical information, including the physiological and anthropometric indicators of the individuals included in this cohort, is summarized in Table 1. The participants were divided into three groups according to their BMI values. The level of SBP, waist-hip ratio, fat mass, body fat percentage and visceral fat area were significantly higher in both overweight and obese individuals than in the control group, with obese participants exhibiting higher values compared with overweight individuals (Kruskal-Wallis test, *P* < 0.001).

**Table 1:** Basic characteristics of the three groups in the study

Variables	Control (*n* = 30)	Overweight (*n* = 26)	Obese (*n* = 34)	*P* value ^b^	Obese vs Overweight ^c^	Obese vs Control ^c^	Overweight vs Control ^c^
Sex (female %), no. (%) ^a^	18 (60.00)	16 (53.33)	14 (46.67)	0.594	–	–	–
Age, year	12.50 ± 0.51	12.73 ± 0.45	12.77 ± 0.47	0.058	0.958	0.072	0.132
BMI, Kg/m^2^	17.49 ± 1.41	23.76 ± 1.00	29.89 ± 3.17	< 0.0001	< 0.0001	< 0.0001	< 0.0001
SBP, mmHg	111.93 ± 9.77	120.27 ± 7.18	123.13 ± 6.23	< 0.0001	< 0.0001	< 0.0001	0.339
DBP, mmHg	68.13 ± 6.77	68.73 ± 4.68	70.07 ± 7.10	0.477	0.69	0.46	0.927
TG, mmol/L	0.88 ± 0.36	1.08 ± 0.67	1.12 ± 0.61	0.207	0.964	0.225	0.342
CHO, mmol/L	4.16 ± 0.76	4.03 ± 0.80	4.34 ± 0.77	0.297	0.268	0.631	0.796
HDL, mmol/L	1.39 ± 0.29	1.24 ± 0.22	1.22 ± 0.20	0.015	0.885	0.018	0.061
LDL, mmol/L	2.32 ± 0.53	2.35 ± 0.61	2.71 ± 0.66	0.031	0.069	0.048	0.987
Waist-hip ratio	0.79 ± 0.03	0.85 ± 0.04	0.91 ± 0.05	< 0.0001	< 0.0001	< 0.0001	< 0.0001
FBG, mmol/L	5.52 ± 0.37	5.56 ± 0.42	5.60 ± 0.42	0.740	0.907	0.718	0.933
Fat mass, Kg	9.00 ± 3.33	19.70 ± 4.05	28.82 ± 6.96	< 0.0001	< 0.0001	< 0.0001	< 0.0001
Body fat percent, %	19.86 ± 6.06	31.63 ± 5.44	38.00 ± 6.60	< 0.0001	< 0.0001	< 0.0001	< 0.0001
Visceral fat area, cm^2^	38.78 ± 14.26	88.76 ± 24.86	138.81 ± 39.78	< 0.0001	< 0.0001	< 0.0001	< 0.0001

Values are given as mean ± SD or number of individuals (%). *BMI* Body mass index, *SBP* systolic pressure, *DBP* Diastolic pressure, *TG* Triglyceride, *CHO* Cholesterol, *HDL* High density lipoprotein, *LDL* Low density lipoprotein, *FBG* Fast blood glucose

^a^*P* value of chi-square test.

^b^*P* value of Kruskal–Wallis test.

^c^*P*-value of Dunn’s post hoc test.

We evaluated both coverage and reproducibility of the non-targeted lipidomic data on our sample. Using Progenesis QI 2.0 and metaX, the non-targeted metabolomics analysis yielded 51,135 positive ion modes (Additional file 2) and 8988 negative ion modes (Additional file 3).

### Overweight and obesity-related features

**Fig. 1:**
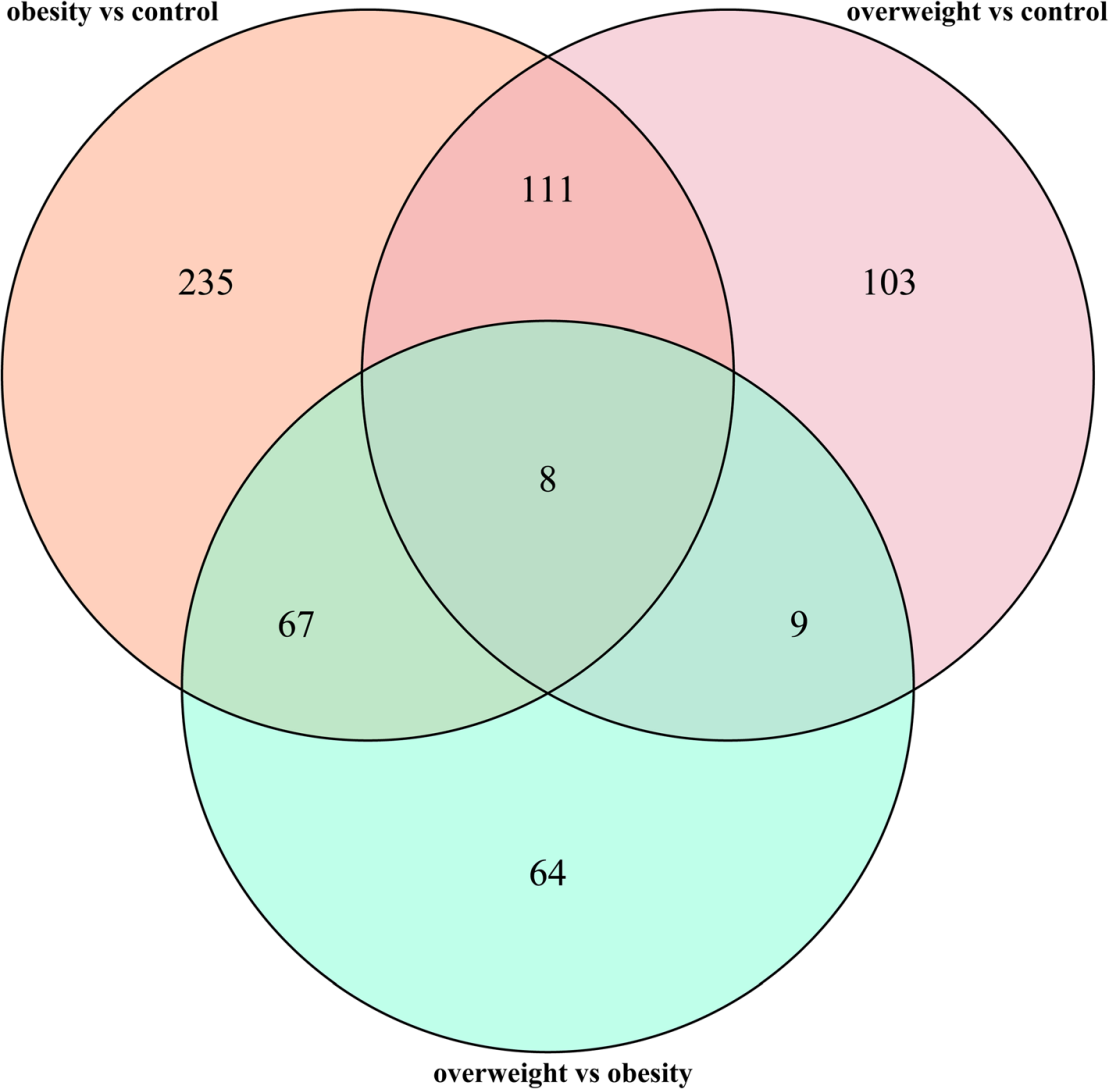
Venn diagram of significant metabolites from the 3 paired comparisons. Venn diagram depicting the number of significant metabolic features from 3 paired comparisons (the direction of change was ignored, *P* < 0.05, Dunn’s post hoc test)

Because of the observed effects in obese adolescents on the lipid profiles, we performed a blocked Kruskal-Wallis test, using the obese group as the blocking factor, followed by Dunn’s hoc test for paired comparisons. As shown in Additional files 4 and 5, 876 positive and 544 negative features were gradually upregulated among the 3 groups. Also, there were 1081 positive and 353 negative features down-regulated in Additional files 6 and 7. Of these, there are lipids or lipid-like compounds, also including organ-oxygen compounds, amino acids, peptides, and analogs, benzyl alcohols, glycerophospholipids and triacylglycerol. As shown in Fig. 1, paired comparisons revealed that 460 features (290 features in Additional file 8 positive and 170 features in Additional file 11 negative) exhibited significant differences between the control and obese group, whereas 231 and 244 features (Additional files 9 and 12, Additional files 10 and 13 in both positive and negative, respectively) showed obvious differences between the overweight versus the control group and the obese group, respectively (*P* < 0.05). Of these significantly changed metabolites, we screened out eight (six positive and two negative) metabolites with significant differences in expression among the three groups. The number of variables distinguishing overweight and obesity suggested that changes in a large fraction of the lipid profiles in overweight and obesity were shared, implying that compared with the control group, the overweight and obese group share similar metabolites.

To quantify the differential features among the 3 groups, all detected features were assessed using criteria: 1) variable importance of the projection (VIP) > 1.0 estimated by partial least squares discriminant analysis (PLS-DA); 2) fold change in mass intensity ≥1.2 or ≤ 0.83; 3) *P* < 0.05.

### Comparison between control and overweight, overweight and obese, and control and obese using random forest classifier and ROC curves

As the qualitative and quantitative analyses revealed significant differences in the metabolites levels among the three groups and indicated a gradual change from control to obese via overweight, we investigated if the metabolites could predict the risk of further obesity development. To assess this possibility, we used a random forest classifier.

**Fig. 2:**
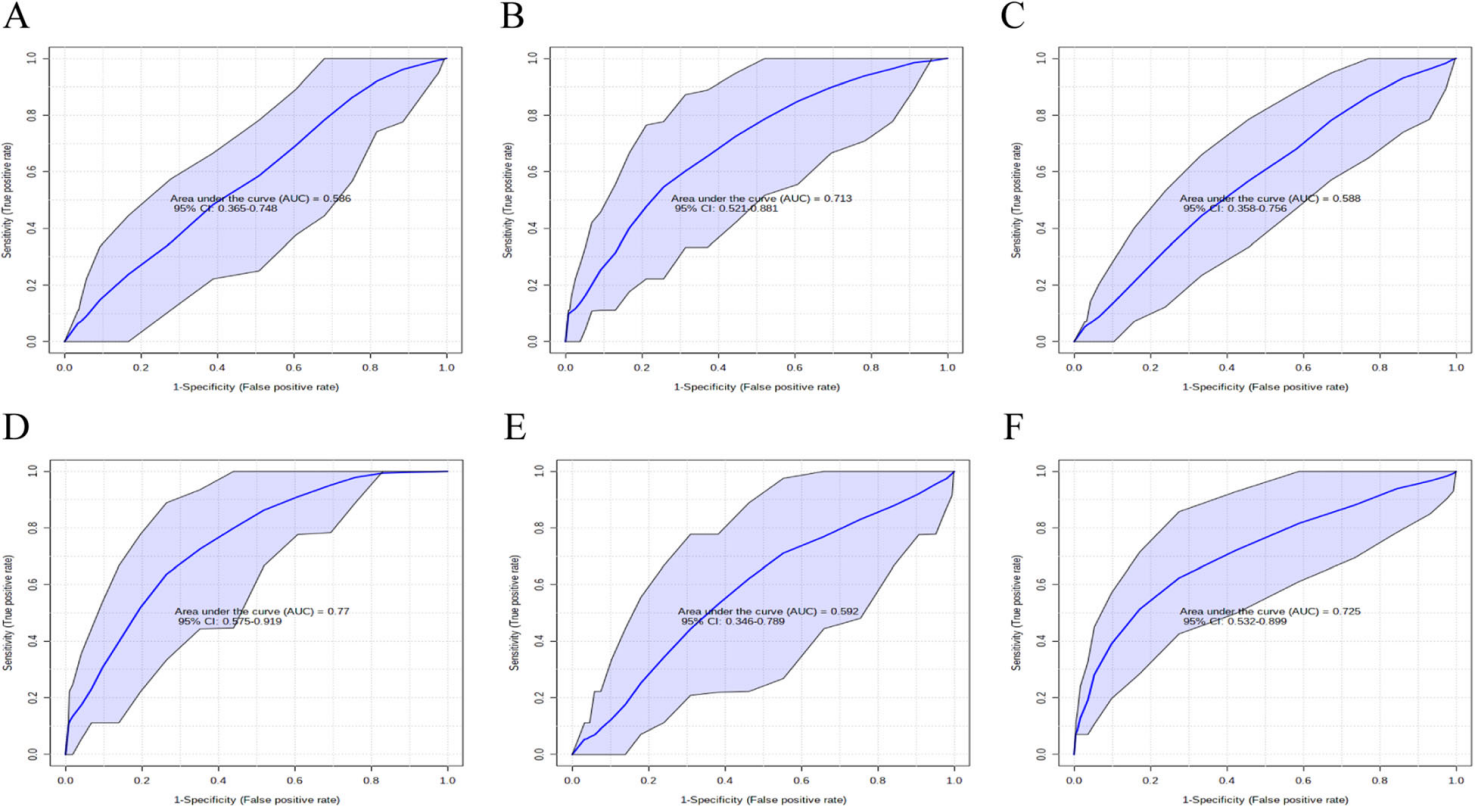
Receiver operating characteristic curves and areas under the ROC curves in the training set. **A-F** ROC and AUC for the validation set with Control and Overweight, Overweight and Obese, and Control and Overweight, respectively. The model was trained using decreased and increased intensity of the detected features from positive and negative ion mode in the training set among control, overweight and obese (*n* = 30)

As illustrated in Fig. 1, 8 metabolites were generated. Based on all the metabolites, the relationships among the three groups were analyzed by the random forest classifier and receiver operating characteristic (ROC) curves. Figure 2A-C shows that the area under the ROC curve (AUC) is 61.90% (95% confidence interval (CI) = 42.00–85.60%), 62.80% (95% CI = 21.50–86.50%), and 74.30% (95% CI = 56.00–91.00%) between control and overweight, overweight and obese, and control and obese in down-regulated both positive and negative ion mode. For up-regulated, the AUC is 59.70% (95% CI = 19.50–82.50%), 65.40% (95% CI = 34.10–75.50%), and 72.10% (95% CI = 49.00–93.50%) in Fig. 2D-F. Together, these results indicate that the lipidomic profiles are regulated in a complex manner during the development of overweight and obesity.

### The level of selected metabolites in the control, overweight and obese groups

**Fig. 3:**
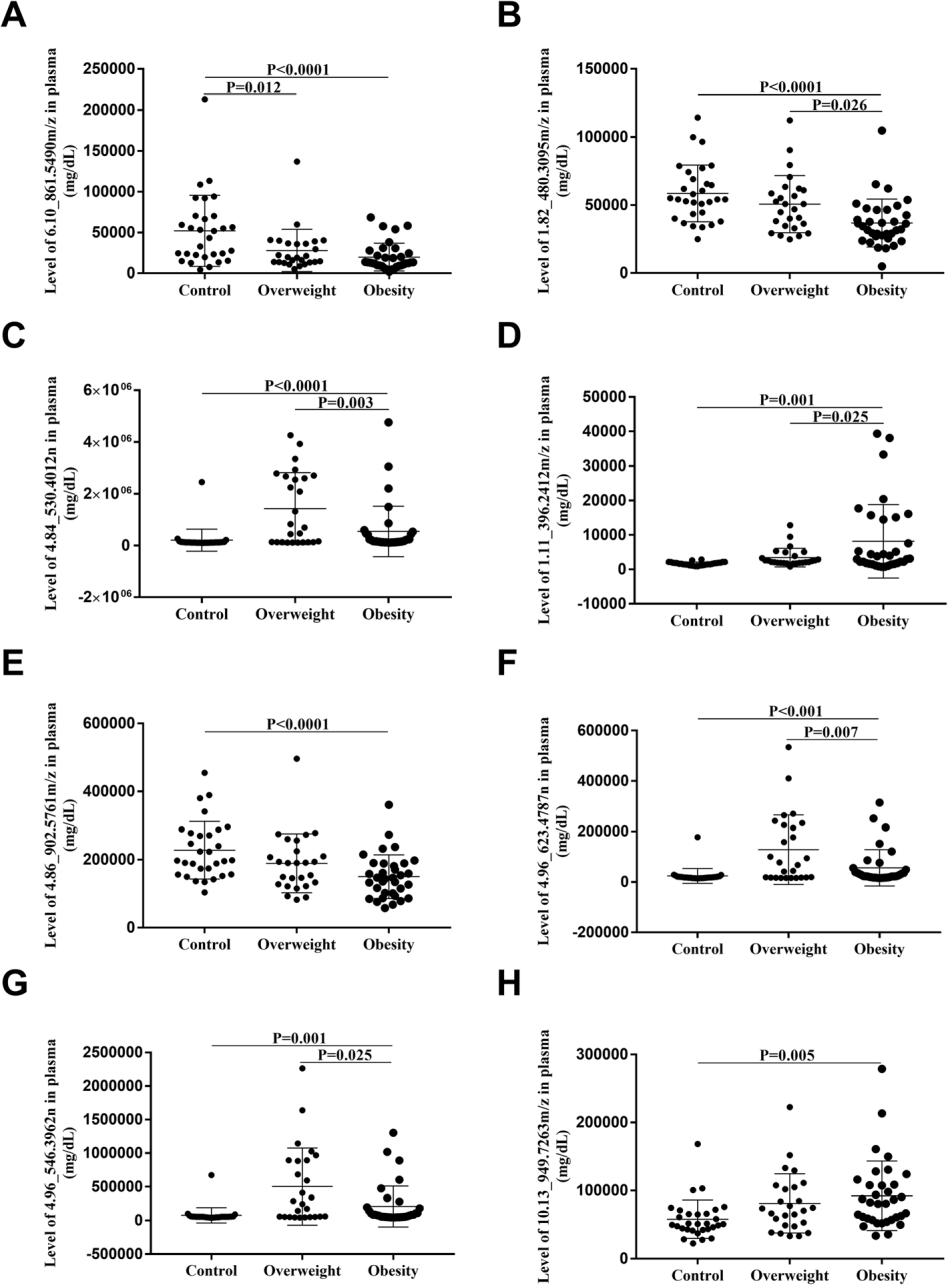
Level of selected metabolites in control, overweight and obese groups. **A** and (**B**) show negative ion modes level in the control, overweight and obese groups. **C-G** show positive ion modes level in the control, overweight and obese groups

As illustrated in Fig. 1, eight metabolites were selected from both positive and negative ion mode lipidomic profiling. The expression of the selected metabolites is shown in Fig. 3. Figure 3A and B indicate that 6.10_861.5490 m/z and 1.82_480.3095 m/z in negative ion mode were gradually decreased in the control, overweight and obese groups. Figure 3D and H exhibit 1.11_396.2412 m/z and 10.13_949.7263 m/z in selected positive ion mode were gradually increased in the control, overweight and obese groups. However, 4.86_902.5761 m/z was gradually decreased in Fig. 3E, 4.84_530.4012n, 4.96_623.4787n and 4.96_546.3962n peaked in the overweight group in Fig. 3C, F and G. In summary, the development of obesity may go through the process of overweight in most cases, but it may directly develop into obesity through the alterations of some lipid metabolites.

### Correlations between the selected metabolites and clinical parameters

**Fig. 4:**
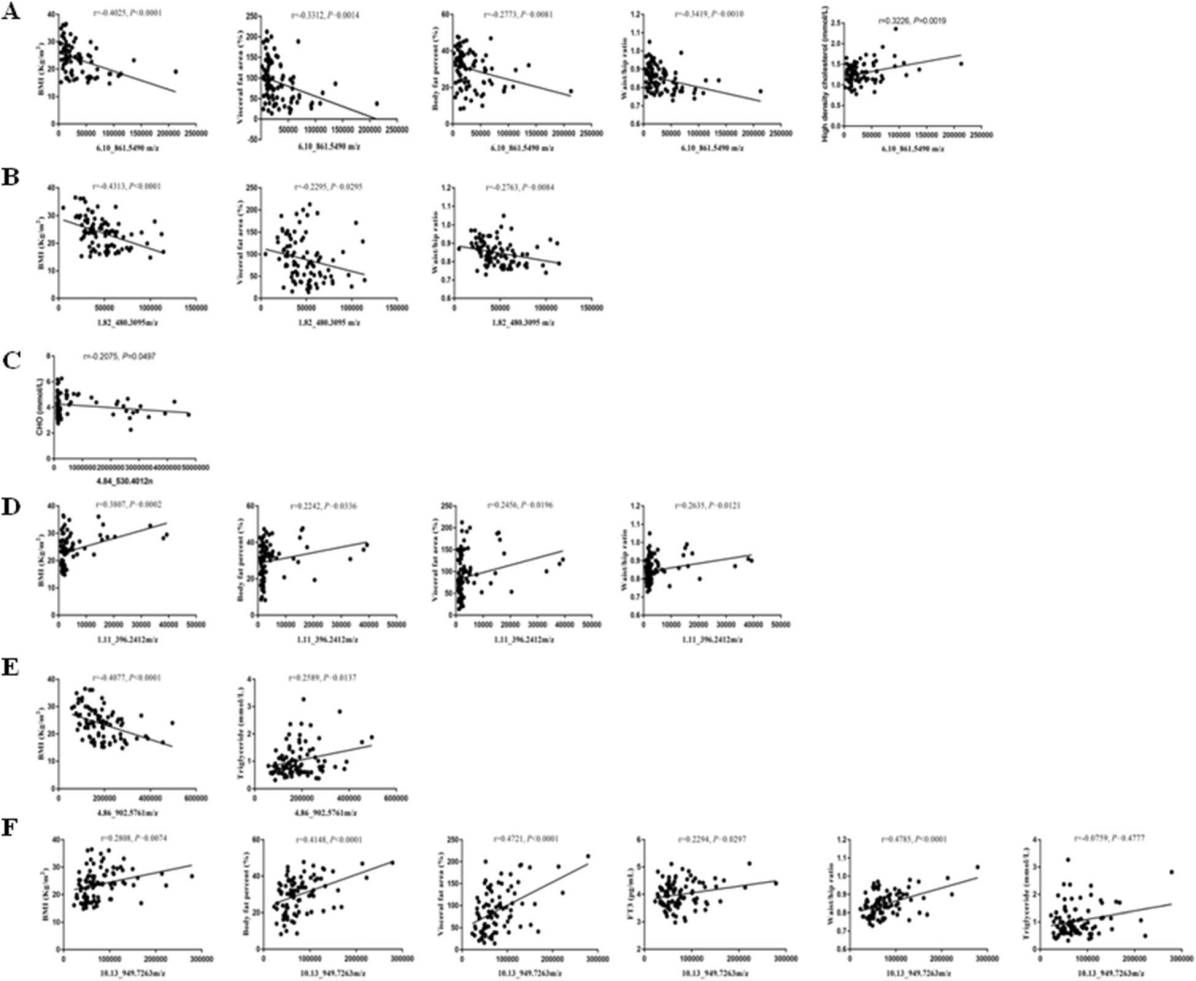
Correlation between clinical parameters and selected features. **A** 6.10_861.5490 m/z was negatively correlated with BMI, visceral fat area, body fat percent, waist/hip ratio, and HDL. **B** 1.82_480.3095 m/z was negatively correlated with BMI, visceral fat area, body fat percent, and waist/hip ratio. **C** 4.84_530.4012n was negatively correlated with cholesterol. **D** 1.11_396.2412 m/z was positively correlated with BMI, visceral fat area, body fat percent, and waist/hip ratio. **E** 4.86_902.5761 m/z was negatively correlated with BMI, but positively with triglyceride. **F** 10.13_949.7263 m/z was positively correlated with BMI, body fat percent, visceral fat area, FT3, waist/hip ratio, and triglyceride

In the body of overweight and obese people, metabolism is inevitably changed. Hence, the metabolites are also changed. To investigate the relationship between the selected metabolites and clinical parameters, we performed a correlation analysis. As shown in Fig. 4A, 6.10_861.5490 m/z was negatively correlated with BMI, visceral fat area, body fat percent, and waist/hip ratio. 1.82_480.3095 m/z was negatively correlated with BMI, visceral fat area, body fat percent, and waist/hip ratio, but positively correlated with triglyceride in Fig. 4B. 4.84_530.4012n was negatively correlated with total cholesterol (CHO) in Fig. 4C. 1.11_396.2412 m/z was positively correlated with BMI, visceral fat area, and waist/hip ratio in Fig. 4D. 4.86_902.5761 m/z was negatively correlated with BMI, but positively with triglyceride (Fig. 4E). 10.13_949.7263 m/z was positively correlated with BMI, visceral fat area, waist/hip ratio, triglyceride, and body fat percent (Fig. 4F).

Phospholipids phosphatidylcholine (PC) and phosphatidylethanolamine (PE) are the two most abundant phospholipid species in eukaryotic cells [18]. Lysophosphatidylcholine (LPC), an important signaling molecule and fatty acid carrier, constitutes 5–20% of total plasma phospholipids [19]. Phosphatidylinositol (PI) plays an important role in cell morphology, metabolic regulation, signal transduction and various physiological functions. 1.82_480.3095 m/z was annotated as PC (15:0/0:0), PE (18:0/0:0), LPC (15:0), and LPE (0:0/18:0). 6.10_861.5490 m/z was annotated as PI (14:0/22:2(13Z, 16Z))- PI (22:2(13Z,16Z)/14:0) (Additional file 3). 1.11_396.2412 m/z was annotated as 18-hydroxycortisol, isohumulinone A, and 11-dihydro-12-norneoquassin; 4.86_902.5761 m/z was annotated as PI (18:0/20:5 (5Z,8Z,11Z,14Z,17Z)); and 10.13_949.7263 m/z was annotated as TG (20:4 (5Z,8Z,11Z,14Z) /20:3(5Z,8Z,11Z) /18:3 (9Z,12Z,15Z)). The levels of TG, 18-hydroxycortisol, isohumulinone A, and 11-dihydro-12-norneoquassin were up-regulated in the obese group, while PC, PE, LPC, LPE, and PI were significantly down-regulated in the obese group than in control and overweight individuals (Additional file 2).

## Discussion

Due to the increased prevalence of obesity in children and adolescents, various studies have been conducted to discover which associations and risk factors increase the likelihood of obesity in children. Although it is still difficult to fully grasp all of the risk factors related to obesity, it is of great significance to control and prevent obesity by combining diet, exercise, physiological factors and psychological factors [2]. The short-term and long-term effects of obesity on children’s health are a major issue due to adverse psychological and health consequences [20]. Potential negative psychological outcomes are depressive symptoms, poor body image, low self-esteem, risk of eating disorders, and behavioral and learning problems; negative health consequences include insulin resistance, type 2 diabetes, asthma, hypertension, and nonalcoholic steatohepatitis [20, 21]. Obese children are more likely to become obese adults, and therefore increase their risk of multiple diseases before they even reach puberty [21].

The characteristics of human lipomics reflect the early stage of lipid metabolism, including pathophysiological changes related to diseases. Wang et al. observed the levels of five LPC species in an obese group were significantly reduced relative to a normal-weight group [22]. In addition, total LPC, LPC18:0, LPC18:2 and LPC20:4 levels in obese and obese subjects with type 2 diabetes were lower than in nonobese adults. There was no difference in the LPC profile between obese individuals and obese subjects with type 2 diabetes [23]. Moreover, Wallace et al. reported several LPC species were associated with BMI and inflammatory markers [24]. Compared with lean subjects, LPC14:0 and LPC18:0 were higher while LPC18:1 was lower in obese subjects [25].

As we all know, obesity can be estimated by several methods: body mass index (BMI), the ratio of weight to the square of height, is used as the most common indicator of obesity [26]. It is convenient and simple, but it can cause changes in cardiovascular and metabolic performance between individuals. However, there are alternative ways to distribute body fat. A higher WHR indicates more intraperitoneal cavity fat and is associated with a higher risk of type 2 diabetes, cardiovascular disease and mortality [27]. At the same time, waist circumference can also be used. Similar to WHR [28], it is considered a more direct and reliable method. Generally, body fat percentage (BFP) is a method used to measure the ratio of adipose tissue to lean mass and water [29], and is usually determined using bioelectrical impedance. BFP is not related to BMI since it is associated with an increase in all-cause mortality, but it is generally suggested to estimate obesity better than BMI [30]. Therefore, this study aimed at Chinese adolescents, a group with a relatively stable diet and lifestyle, carried out a lipidomic study to observe the development process of obesity and to screen out some biochemical indicators for predicting obesity.

In the present study, the levels of TG, 18-hydroxycortisol, isohumulinone A, and 11-dihydro-12-norneoquassin were up-regulated in the obese group, while PC, PE, LPC, LPE, and PI were significantly down-regulated in the obese group relative to the control and overweight individuals. 1.82_480.3095 m/z was annotated as PC (15:0/0:0), PE (18:0/0:0), LPC (15:0), and LPE (0:0/18:0). 6.10_861.5490 m/z was annotated as PI (14:0/22:2(13Z, 16Z)) - PI (22:2(13Z, 16Z)/14:0) (Additional file 3). According to Fig. 1, eight metabolites generated only in 1.11_396.2412 m/z were annotated as 18-hydroxycortisol, isohumulinone A, and 11-dihydro-12-norneoquassin; 4.86_902.5761 m/z was annotated as PI (18:0/20:5 (5Z,8Z,11Z,14Z,17Z)); and 10.13_949.7263 m/z was annotated as TG (20:4 (5Z,8Z,11Z,14Z) /20:3(5Z,8Z,11Z) /18:3 (9Z,12Z,15Z)) (Additional file 2). These data suggest that the development of obesity does not always have to go through an overweight stage, and it may develop directly due to some changes in lipid metabolism.

There are also some limitations to our study. Firstly, it was a cross-sectional study that only addressed the alterations of lipidomic profiling in normal, overweight and obese students. Furthermore, the subjects were just grouped according to BMI rather than randomly, and therefore, this may produce selection bias. In addition, this is a small sample study. So based on the above limitations, more large-scale population studies are needed for future investigations.

## Conclusions

In conclusion, this investigation identified eight altered metabolites in Chinese obese and overweight students. These discriminatory metabolites may play important roles in the pathogenesis of obesity and provide a basis for evaluating the risk of and monitoring obesity development.

## Supplementary Information

**Additional file 1.** Basic information, clinical and phenotypic data of participants.

**Additional file 2.** 51135 positive ion modes from non-targeted metabolomics analysis.

**Additional file 3.** 8988 negative ion modes from non-targeted metabolomics analysis.

**Additional file 4.** Up-regulated positive features among the 3 groups.

**Additional file 5.** Up-regulated negative features among the 3 groups.

**Additional file 6.** Down-regulated positive features among the 3 groups.

**Additional file 7.** Down-regulated negative features among the 3 groups.

**Additional file 8.** Different positive features between the control and obese group.

**Additional file 9.** Different positive features between the control and overweight group.

**Additional file 10.** Different positive features between the obese and overweight group.

**Additional file 11.** Different negative features between the control and obese group.

**Additional file 12.** Different negative features between the control and overweight group.

**Additional file 13.** Different negative features between the obese and overweight group.

## Data Availability

The author has produced the original data described in this manuscript, which can be obtained free of charge by any scientist who wants to use it, without violating the confidentiality rules of the participants.

## Abbreviations

BMI: Body mass index
SBP: Systolic pressure
DBP: Diastolic pressure
TG: Triglyceride
CHO: Cholesterol
HDL: High density lipoprotein
LDL: LOW density lipoprotein
FBG: Fast blood glucose
PC: Phosphatidylcholine
PE: Phosphatidylethanolamine
PI: Phosphatidylinositol
DDA: Data-dependent acquisition
DIA: Data-independent acquisition
LC-MS/MS: Liquid chromatography-tandem mass spectrometry
MS: Mass spectrometry
UPLC-MS: Ultra-performance liquid chromatography–mass spectrometry
VIP: Variable importance of the projection
